# Web-Based Interventions for Dietary Behavior in Adults With Type 2 Diabetes: Systematic Review of Randomized Controlled Trials

**DOI:** 10.2196/16437

**Published:** 2020-08-28

**Authors:** Jedha Dening, Sheikh Mohammed Shariful Islam, Elena George, Ralph Maddison

**Affiliations:** 1 Institute for Physical Activity and Nutrition School of Exercise and Nutrition Sciences Deakin University Geelong, Victoria Australia

**Keywords:** type 2 diabetes, dietary behavior, diet, glycemic control, self-management, eHealth, web-based, HbA_1c_

## Abstract

**Background:**

Type 2 diabetes mellitus (T2DM) is among the most prevalent noncommunicable health conditions worldwide, affecting over 500 million people globally. Diet is a key aspect of T2DM management with dietary modification shown to elicit clinically meaningful outcomes such as improved glycemic control, and reductions in weight and cardiovascular disease risk factors. Web-based interventions provide a potentially convenient and accessible method for delivering dietary education, but its effects on dietary behavior in people with T2DM are unknown.

**Objective:**

The objective of this review was to determine the effectiveness of web-based interventions on dietary behavior change and glycemic control in people with T2DM.

**Methods:**

Per PRISMA (Preferred Reporting Items for Systematic Reviews and Meta-Analyses) guidelines, systematic literature searches were performed using Medline, Embase, The Cochrane Library, and CINAHL to retrieve papers from January 2013 to May 2019. Randomized controlled trials of web-based interventions in adults with T2DM with reported dietary assessment were included. Population and intervention characteristics, dietary guidelines and assessments, and significant clinical outcomes were extracted. Differences between groups and within groups were assessed for dietary behavior and clinical outcomes.

**Results:**

There were 714 records screened, and five studies comprising 1056 adults were included. Studies measured dietary changes by assessing overall diet quality, changes in specific dietary components, or dietary knowledge scores. Significant improvements in dietary behavior were reported in four out of the five studies, representing healthier food choices, improvements in eating habits, reductions in carbohydrates, added sugar, sodium, saturated fat and overall fat intake, and/or increases in dietary knowledge. Three studies found significant mean reductions for hemoglobin A1c ranging from –0.3% to –0.8%, and/or weight ranging from –2.3 kg to –12.7 kg, fasting blood glucose (–1 mmol/L), waist circumference (–1 cm), and triglycerides (–60.1 mg/dL). These studies provided varied dietary recommendations from standard dietary guidelines, national health program guidelines, and a very low carbohydrate ketogenic diet.

**Conclusions:**

This review provided evidence that web-based interventions may be an effective way to support dietary behavior change in people with T2DM, potentially leading to changes in glycemic control and other clinical outcomes. However, the evidence should be viewed as preliminary as there were only five studies included with considerable heterogeneity in terms of the diets recommended, the dietary assessment measures used, the complexity of the interventions, and the modes and methods of delivery.

## Introduction

Type 2 diabetes mellitus (T2DM) is among the most prevalent noncommunicable diseases worldwide, estimated in 2018 to affect more than 500 million people across 45 countries [1]. T2DM is a metabolic disorder characterized by hyperglycemia; therefore, obtaining glycemic control is the overarching goal of T2DM treatment [2]. Glycemic control is generally defined as a hemoglobin A_1c_ (HbA_1c_) level of 7% or less. However, it is recognized that tighter glycemic control of 6.5% or less may further reduce the risk of macro and microvascular complications such as cardiovascular disease (CVD), neuropathy, nephropathy, and retinopathy [2], along with decreasing the risk of mortality [3]. The American Diabetes Association (ADA) [2] recommends education aimed at lifestyle modifications, including diet and physical activity, and prescription of medications as necessary for the management of T2DM.

It is well recognized that improving diet can optimize glycemic control, with research demonstrating improved diet quality can reduce HbA_1c_ to a similar or greater level than the provision of medication to patients with T2DM [2,4]. Even where medication is necessary, diet remains an important component of the overall treatment plan for people with T2DM [2]. Additionally, improved diet quality may optimize weight management, blood pressure, and lipid profile, which in turn may decrease the risk of CVD and stroke in people with T2DM [4]. Previous qualitative studies including people with T2DM, have confirmed that dietary support is one of their leading preferences for self-management education [5-8]. Nevertheless, there remains a substantial gap between the need for and the provision of dietary education for those with T2DM. Initial and ongoing diabetes education is the role of an individuals’ multidisciplinary care team and/or provided by health care professionals or organizations through structured diabetes education programs [9]. However, rates of receiving any type of diabetes education are reported to be low globally, 23-66% in the United States [10], 11% in the United Kingdom [11], and 40% in Australia [9]. One explanation is access to and availability of health care professionals [6,12], the number of people with T2DM outweighing the number of health care professionals available to provide diabetes education [12]. Additionally, the cost and labor of delivering diabetes education programs face-to-face represent a significant challenge to organizations [13,14].

For the past two decades, researchers have become increasingly interested in providing diabetes education via technological means, as it represents a delivery method that has greater reach and access for people with T2DM [15], and is potentially more cost-effective [16]. Compared to usual care, web-based programs in people with T2DM have been shown to reduce HbA_1c_ by 0.47%-1.49% [17], while mobile health (mHealth) interventions reduced HbA_1c_ by an average of 0.8% [18]. Interventions using mobile apps show reductions in HbA_1c_ of 0.4%-1.9% [19], and the provision of telehealth has been associated with an average HbA_1c_ reduction of 0.17% [20]. In terms of the influence of intervention features on HbA_1c_ outcomes in people with T2DM, when compared to mHealth and telehealth interventions, statistically significant results were only found for web-based interventions, which may indicate that web-based interventions are particularly useful for eliciting behavior change in people with T2DM [21].

Although people with T2DM need and want dietary education, web-based interventions to date have overwhelmingly focused on overall self-management [10,21,22]. While some interventions have included a healthy eating component within the intervention package, assessment of dietary adherence or behavior remains scarce. To date, we are not aware of any review that has investigated the effects of web-based interventions on change in dietary behavior in people with T2DM. Therefore, the primary aim of this systematic review was to identify and synthesize the available evidence from randomized controlled trials (RCTs) and determine the effectiveness of web-based interventions on dietary behavior change and glycemic control in people with T2DM.

## Methods

This systematic review was conducted per the PRISMA (Preferred Reporting Items for Systematic Reviews and Meta-Analyses) guidelines [23]. The review protocol was registered at PROSPERO (International Prospective Register of Systematic Reviews) (2018 #CRD42018109312).

### Eligibility Criteria

Web-based interventions were included and defined as web-based if participants received information and directly interfaced with the internet, but they were not required to input data to a website [24]. In addition, studies were included if they were published in English; were RCTs or pilot RCTs; included an assessment of nutrition, diet, or dietary behavior; included adult participants (≥18 years) with diagnosed T2DM. Exclusion criteria were studies using non–web-based digital interventions; studies including participants with prediabetes or type 1 diabetes; studies including a combination of T2DM and participants with other types of diabetes or where outcomes for multiple chronic diseases were assessed; and studies focused on diabetes prevention.

### Information Sources

A systematic literature search of four electronic databases, Medline, Embase, The Cochrane Library, and CINAHL for relevant papers published between January 2013 and May 2019, was conducted. Papers published before January 2013 were excluded as previous systematic reviews have reported on web-based, computer-based, and digital interventions in people with T2DM up to this date [22,25,26]. From these reviews, we extracted papers that met our inclusion criteria. Additionally, we conducted an in-depth exploration of reference lists for related papers and searched grey literature, including Google Scholar.

### Search Strategy

Keywords used in the search were (“Type 2 Diabet*” OR “diabetes mellitus, type 2” OR “T2DM” OR “T2D”) AND (“web-based” OR “internet” OR “online” OR “digital” OR “information technology” OR “IT” OR “computer-assisted” OR “computer-based” OR “computer interface” OR “ehealth”) AND (“diet*” OR “nutrition” OR “self-management” OR “lifestyle modification”).

### Data Extraction

Search results were merged using reference management software (Endnote 8) and duplicate papers removed. Screening of the titles and abstracts for individual studies were conducted in duplicate, independently, by two authors (JD, SI) with disagreements resolved by consensus. Articles deemed eligible for full-text review were assessed for eligibility by two authors, independently (JD, SI), and disagreements for inclusion were reached via consensus. Corresponding papers from the same study were merged to extract relevant outcomes. The following parameters were extracted from included studies: author/date, study design, sample size, total study period and length of follow up, population characteristics (including location, age, and comorbidities), behavioral change theory or model, digital intervention characteristics (including the type of digital intervention and duration of exposure), intervention providers, type of dietary intervention or guidelines administered, the dietary assessment used, dietary behavior change outcomes, and significant T2DM clinical outcomes including glycemic control as indicated by blood glucose levels or HbA_1c_, and biomarkers weight, waist circumference, and CVD biomarkers. We also extracted intervention components, website usability rates, modes and methods of delivery, and process evaluation measures.

### Assessment of Study Risk of Bias

The risk of bias was assessed by two researchers independently using the Cochrane risk-of-bias tool for randomized trials (RoB 2) [27]. RoB 2 provides an in-depth framework structured into five domains to assess the risk of bias in RCTs. These five domains assess the risk of bias arising from the randomization process, deviations from intended interventions, missing outcome data, outcome measurements, and selection of reported results. During the assessment, each domain is given a rating of low, high, or unclear. Conflicting assessment ratings were resolved by consensus. Results were then calculated to reach a quality assessment rating of poor, fair, or good quality.

### Data Analysis

For qualitative analysis, differences in end intervention measures between groups and change between groups were reported, depending on the outcomes reported for individual studies. We also reported significant within-group changes. Data were considered statistically significant if the reported *P* value was <.05.

## Results

**Figure 1:**
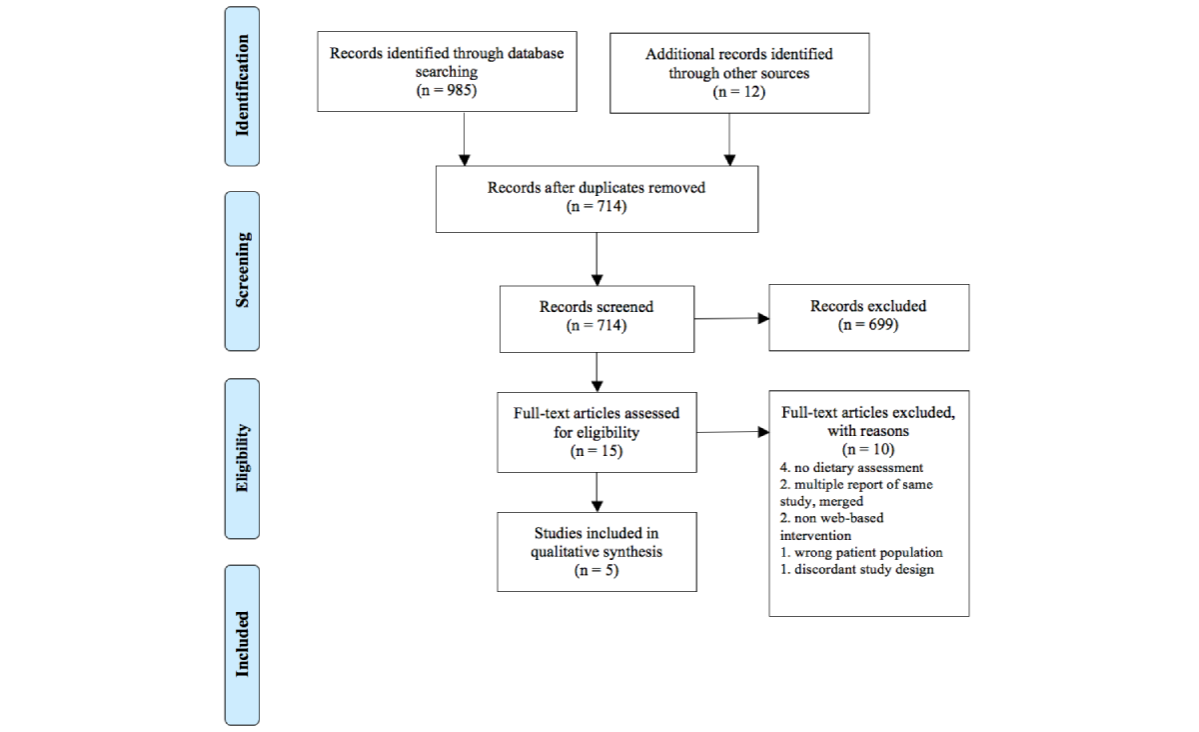
Prisma Flow Chart.

The search identified a total of 714 papers, of which 15 full-text articles were assessed for eligibility, and five studies met the inclusion criteria and were included in this review (Figure 1) [28-32]. Corresponding papers from the same studies [33-37] provided supporting information extracted for this review. Four of the studies reviewed were RCTs, and one was a pilot RCT. Each study described a web-based intervention where participants received information that directly interfaced with the internet and included some form of dietary assessment. All the studies included only adults with T2DM; a summary of the included studies is shown in Table 1.

**Table 1:** Summary of study characteristics for included randomized controlled trials.

Author/date	Study design	Sample size, N	Population characteristics	Theory/model	Intervention participants, N, program name, digital intervention characteristics	Type of diets prescribed	Intervention/follow-up period
Ramadas et al, 2018 [29]	RCT^a^	128	Adults with T2DM^b^, mean age 50.5 years, most with a family history of T2DM. Malaysia	Transtheoretical Model Stages of Change, user-centered design	N=62, myDIDeA^c^, received personalized intensive dietary intervention via website + standard care	IG^d^: standard dietary guidelines CG^e^: no prescribed diet	6-month intervention, 12-month follow-up
Hansel et al, 2017 [28]	RCT	120	Adults with abdominal obesity and T2DM, mean age 57 years. Paris	No theory reported	N=60, ANODE^f^, fully automated web-based nutritional support program	IG: National Nutrition & Health Program guidelines CG: received general diet advice	16-week intervention
Saslow et al, 2017 [30]	Pilot RCT	25	Overweight adults with T2DM, mean age 55.6 years. California	No theory reported	N=12, dietary instruction provided via email lessons including mindfulness training and lifestyle advice	IG: VLKCD^g^ (20-50 g/carbs per day) CG: ADA^h^ “create your plate” diet	16-week intervention, 32-week follow-up
Glasgow et al, 2003 [31]	RCT	320	Adults with T2DM, mean age 59 years. Colorado	Self-efficacy theory, social support theory	N=N/A^i^, D-Net^j^, two intervention arms: tailored self-management training, peer support	All groups received general healthy eating advice to decrease fat and increase fruit and vegetable intake	3-month intervention, 10-month follow-up
Glasgow et al, 2012 [32]	RCT	463	Adults with T2DM, mean age 58.4 years. Colorado	Social-ecological theory, social cognitive theory and the “5 As” self-management model	N=189, two intervention arms: CASM^k^: self-administered computer-assisted self-management; N=182, CASM+^l^ with enhanced social support	All groups received general advice to decrease fat and eat a healthy diet	4-month intervention, 12-month follow-up

^a^RCT: randomized controlled trial.

^b^T2DM: type 2 diabetes mellitus.

^c^myDIDeA: Malaysian Dietary Intervention for People with Type 2 Diabetes: An e-Approach.

^d^IG: intervention group.

^e^CG: control group.

^f^ANODE: Accompagnement Nutritionnel de l’Obésité et du Diabète par E­coaching.

^g^VLCKD: very low carbohydrate ketogenic diet.

^h^ADA: American Diabetes Association.

^i^N/A: not applicable.

^j^D-Net: Diabetes Network.

^k^CASM: computer-assisted self-management.

^l^CASM+: computer-assisted self-management plus social support.

### Participants and Intervention Characteristics

The included studies provided results from a total of 1056 participants representing three countries—France [28], Malaysia [29], and the United States [30-32]. The number of participants ranged from ≤26 in the pilot study [30] to ≤463 in the RCTs [32]. The mean age of participants ranged from 50.5 years [29] to 59 years [32]. Three studies had an intervention period of 4 months [28,30,32], one was 3 months [31], and one was 6 months [29]. Two studies included a 12-month follow up [29,32], one had a 10-month follow up [31], and another had a 32-week follow up [30]. The Malaysian Dietary Intervention for People with Type 2 Diabetes: An e-Approach (myDIDeA) [29] focused solely on dietary behavior by providing participants with a personalized intensive dietary intervention. The remaining studies assessed dietary behavior but included dietary recommendations alongside physical activity [28,30], mindfulness training [30], social support and self-management information [31,32], and face-to-face contact [32]. Three studies included health practitioner-assistance [29,31,32], ranging from contact with a nutritionist [29,32] through to availability of health care coaches and professionals [31,32], and physicians [32]. The Accompagnement Nutritionnel de l’Obésité et du Diabète par E­coaching (ANODE) study [28] was a fully automated intervention aside from providing technical assistance. Three studies [29,31,32] utilized behavioral theories or models, which varied widely. Only myDIDeA [29] applied user-centered design theory to support intervention development.

### Dietary Recommendations and Measurement

Three of the studies indicated the type of dietary recommendations prescribed [28-30], which were standard diabetes dietary guidelines modified to suit the Malaysian population [29], the National Nutrition and Health Program guidelines of France [28], and a very low carbohydrate ketogenic diet (VLCKD) [30]. The remaining two studies, computer-assisted self-management/computer-assisted self-management plus social support (CASM/+) [32] and the Diabetes Network (D-Net) [31], provided self-administered and/or personalized tailored dietary instruction from health professionals with general healthy eating recommendations and goal setting to reduce fast foods, fried foods, or sugar-sweetened beverages, and increase fruit and vegetable consumption. The tools and scales used for dietary assessment varied widely across all five studies (Table 2). myDIDeA [29] used a validated Malaysian 36-item Dietary Knowledge, Attitude, and Behavior (DKAB) score. ANODE [28] used the International Diet Quality Index (DQI-I) with food frequency questionnaires from both 24-hour and 3-day diet recalls. CASM/+ [32] used dietary questionnaires measured by two validated scales, the 20-item Kristal Fat and Fiber Behavior scale (FFB) and the 15-item Block/National Cancer Institute (NCI) Fat Screener. D-Net [31] also used the Block/NCI Fat Screener along with the 8-item “Starting the Conversation” scale. The VLCKD [30] used unvalidated self-reported dietary intake obtained from participants’ entries on a consumer-based website.

**Table 2:** An overview of dietary behavioral outcomes.

Reference	Dietary assessment	Baseline dietary assessment results	Postintervention dietary changes
Ramadas et al, 2018 [29]	DKAB^a^ score, DSOC^b^	IG^c^: DKAB 34.2 (5.2), DSOC 193.3 (14.6) CG^d^: DKAB 33.7 (5.5), DSOC 191.2 (16.2)	IG: DKAB 54.0 (8.7), DSOC 199.7 (18.2) CG:DKAB 41.3 (7.7), DSOC 191.5 (15.1) DKAB score significantly improved in both groups with the margin of improvement higher in the IG, a difference of 12.2 points between groups. No difference between groups in DSOC at 6 months. IG showed improved DCOS at 12-month follow-up and no change in CG
Hansel et al, 2017 [28]	DQI-I^e^	IG: DQI**-**I 54.0 CG: DQI**-**I 52.8	IG: Significant increase in the DQI-I score of 4.55, total 58.55 CG: Decrease in DQI-I score of –1.68, total 51.12 Dietary changes towards healthier foods were noted in the IG, particularly for saturated fat (*P*=.02) and sodium (*P*<.001)
Saslow et al, 2017 [30]	Self-reported dietary intake (MyFitnessPal) and self-reported subjective experience of diets	IG: nonfiber carbohydrates (g) 163.6 (86.7), fat (g) 77.1 (41.4), protein (g) 83.3 (18.0), sugar (g) 50.6 (33.8) CG: nonfiber carbohydrates (g) 152.0 (58.9), fat (g) 81.3 (27.3), protein (g) 74.5 (17.2), sugar (g) 44.9 (23.8)	Self-reported dietary intake showed the IG ate fewer grams of nonfiber carbohydrates and sugar compared to CG. No differences in protein and fat between groups. Change in mean carbohydrate intake in IG from 39.6% of calories to 15.5%. Compared to CG, IG rated themselves as less likely to cheat on their diet, with a large effect size of at least Cohen *d*=–1.0
Glasgow et al, 2003 [31]	FFB^f^ and the NCI^g^ Fat Screener	Not reported	Trending improvements in FFB in both IGs but no significant differences between groups
Glasgow et al, 2012 [32]	“Starting the Conversation” scale and NCI Fat Screener	IG: eating habits 2.18 (0.2), fat intake 34.86 (28) CG: eating habits 2.13 (0.3), fat intake 35.18 (40)	IG: eating habits 2.32 (0.2), fat intake 33.22 (24) CG: eating habits 2.23 (0.3), fat intake 33.91 (37) The combined IG CASM^h^/CASM+^i^ significantly improved eating habits more than CG over 12 months (chi-square = 9.01), fat intake (chi-square = 6.01)

^a^DKAB: Dietary Knowledge, Attitude, and Behavior.

^b^DSOC: Dietary Stages of Change.

^c^IG: intervention group.

^d^CG: control group.

^e^DQI-I: International Diet Quality Index.

^f^FFB: Kristal Fat and Fiber Behavior scale.

^g^NCI: National Cancer Institute.

^h^CASM: computer-assisted self-management.

^i^CASM+: computer-assisted self-management plus social support.

### Dietary Behavior Change and Clinical Outcomes

Four of the studies reported a statistically significant dietary behavior change in the intervention group (Table 2) [28-30,32]. Compared to a control group receiving usual care, ANODE [28] found significant improvements in dietary quality with participants choosing healthier foods overall and improvements in saturated fat and sodium intake. Similarly, compared to an enhanced care control group, CASM/+ [32] found that participants’ overall eating habits improved along with reductions in overall fat intake. Compared to a control group prescribed the ADA’s “create-your-plate” diet, participants following a VLCKD [30] demonstrated adherence with decreased consumption of carbohydrates within the prescribed range of 20-50 grams per day and decreased consumption of added sugar. Compared to a control group prescribed usual care, myDIDeA [29] showed that web-based interventions could be a feasible option for supporting dietary behavior change for people with T2DM in developing countries such as Malaysia, with participants in the intervention group achieving a 12.2-point difference in DKAB score. CASM/+ [32] demonstrated dietary behavior change in individuals with lower literacy and numeracy and diverse and higher-risk populations such as American Indian/Alaska Native, Asian, Black and African American, and Latino.

Changes in clinical outcomes were inconsistent and differed across studies (Table 3). Two studies reported statistically significant improvements in glycemic control for HbA_1c_ between groups compared to usual care [28] and the ADA’s “create-your-plate” diet [30]. One study found significant between-group differences in fasting blood glucose and HbA_1c_ [29] compared to usual care, benefits that were only observed in the intervention group at 12-months follow-up. However, clinical outcomes were not reflected across all four studies that reported significant changes in dietary behavior. CASM/+ [32] reported no significant clinical improvements. Reductions in weight or waist circumference were seen in two studies [28,30]. Twenty percent of the ANODE study’s [28] intervention participants achieved >5% weight loss, and 90% achieved at least 5% weight loss in the VLCKD intervention [30]. Weight reductions could be explained by improved overall diet quality and decreased fat intake [28], decreased sugar and carbohydrate consumption [30], greater calorie deficits in both intervention groups compared to control groups, along with recommendations for physical activity included in both interventions. Additionally, following a VLCKD yielded significant reductions in triglycerides [30].

**Table 3:** Significant clinical outcomes for dietary intervention groups. Data were considered statistically significant if *P*<.05.

Author/date/reported mean and outcomes measured			Baseline		Timepoint		Outcome		Within-group changes (*P* value)		Between-group changes (*P* value)
**Ramadas et al, 2018 [29], mean (SD)**											
	HbA_1c_^a^ (%)	9.1 (2.0)		6 months = 8.7 (1.9)		12 months = 8.5 (1.8)		.004		—^b^	
	Fasting blood glucose (mmol/L)	8.9 (3.9)		6 months = 8.1 (2.7)		12 months = 7.9 (2.5)		.015		—	
**Hansel et al, 2017 [28], mean (SD)**											
	HbA_1c_ (%)	7.16 (0.78)		—		6.86 (0.94)		—		<.001	
	Weight (kg)	93.3 (16.2)		—		91 (3.0)		—		.01	
	Waist circumference (cm)	110 (10)		—		109.1 (4.7)		—		.01	
**Saslow et al, 2017 [30], mean (SD) and mean (EMM^c^)**											
	HbA_1c_ (%)	7.1 (0.4)		16 weeks = 6.2 (–1.1, –0.6)		32 weeks = 6.3 (–1.1, –0.6)		—		.002	
	Weight (kg)	109 (24.9)		16 weeks = 100.5 (–11.9, -5.2)		32 weeks = 96.3 (–7.3, 1.3)		—		<.001	
	Triglycerides (mg/dL)	183 (135)		16 weeks = 147.5 (–65.7, –5.2 EMM)		32 weeks = 122.9 (–46.0, 33.6 EMM)		—		.01	

^a^HbA_1c_: hemoglobin A_1c_

^b^Not applicable

^c^EMM: estimated marginal means.

### Attrition, Website Features, and Usability

An overview of intervention components, attrition, usability, and modes and methods used to deliver the interventions is shown in Multimedia Appendix 1. The attrition rate varied among studies, with the highest dropout rate seen in the CASM/+ interventions [32], losing 34.2% of intervention participants randomized to two complex intervention arms, compared to 19.5% of the control group. The lowest attrition rate was seen in myDIDeA, losing 4.8% of intervention participants and 10.6% of the control group, perhaps because the intervention focused solely on providing a structured dietary intervention and implemented a user-centered approach. Across all web-based interventions, the highest website usage was reported in the first month, followed by declined usage over time. Three studies reported login rates as a measure for usability [28,31,32]. Only one study [29] reported both login rates and time spent on site. Various modes and methods of delivering intervention components were reported, including providing content on a website, which was used in four of the five studies [28,29,31,32], while one sent content via email with text, videos, and links to various web resources [30]. Four studies provided some form of self-monitoring and feedback, whether automated or assisted by a health care professional [28,29,31,32]. Three of the five interventions [29-31] provided updated intervention materials to participants ranging from biweekly to bimonthly. No associations could be drawn between these methods and participant adherence and intervention outcomes due to heterogeneity in the methods of delivery and a lack of detailed reporting.

### Postintervention Process Evaluation

Postintervention process evaluation measured levels of adherence, usability, acceptability, and program satisfaction (Multimedia Appendix 2). The most common statistic used to measure adherence or intervention usage was website login rates [28,29,31,32], with studies consistently demonstrating higher login rates early in the intervention (Multimedia Appendix 1). Two studies included a more comprehensive process evaluation [28,29], both of which provided participants with self-reported feedback questionnaires. myDIDeA [29] usability rates were 72%, acceptability 62%, and program satisfaction 64%. ANODE’s [28] satisfaction rate was 70%. D-Net [31] indicated an implementation percentage of 100% related to their dietary assessment component but did not provide overall satisfaction rates.

### Overall Study Quality

**Figure 2:**
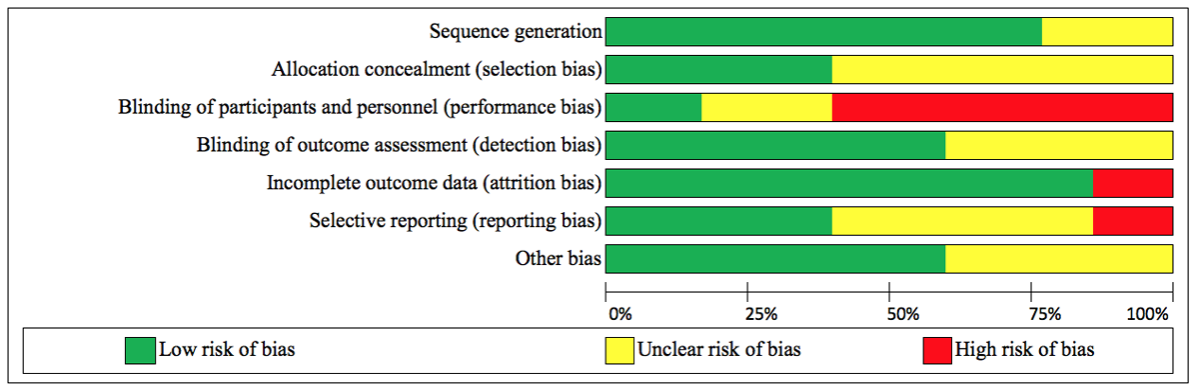
Risk of bias. Judgments about each risk of bias item presented as percentages across all included studies.

Figure 2 summarizes the risk-of-bias assessment [27]. Four studies had a reasonable sequence generation described [28,30-32]. It was unclear in three studies if allocation concealment was adequate [29,30,32]. Only one study sufficiently blinded participants and personnel [29], one study was unclear [31], while the remainder did not blind [28,30,32]. However, blinding in dietary behavior studies is often not feasible. Three studies sufficiently blinded outcome assessors [28-30], while two remained unclear [31,32]. Only one study failed to provide complete outcome data [30]. Two studies reported full datasets [28,31], two were unclear [30,32], while one [29] had several outcomes included in study protocols [36] that were missing from the final study outcomes. Other biases were low [28,31,32] or unclear [29,30]. Based on these assessments, only one study [28] was assessed to be of fair quality, while the remaining four were assessed to be of poor quality.

## Discussion

This systematic review is the first to demonstrate the potential for web-based interventions to achieve significant improvements in dietary behavior in people with T2DM. Moreover, this review showed that improved glycemic control could be achieved using web-based dietary interventions. However, improvements in dietary behavior did not consistently result in improvements in glycemic control across studies.

One explanation for this inconsistency may be the varied dietary recommendations provided to participants across the studies (Table 1). While it is recognized that various dietary patterns are suitable for the management of T2DM [2], it appears that providing participants with more direct dietary recommendations may facilitate greater clinical outcomes. The ANODE study prescribed the National Nutrition and Health Program guidelines of France, myDIDeA prescribed evidence-based standard diabetes dietary guidelines modified to suit the Malaysian population, and the VLCKD prescribed specific guidelines for carbohydrate consumption. In contrast, CASM/+ only provided participants with general healthy eating information. Another explanation may be intervention complexity, as web-based dietary interventions in this review varied widely in terms of the number of components provided to participants (Multimedia Appendix 1). Previous literature [6,38] indicated overly complex interventions might provoke a lack of motivation due to confusion, provision of irrelevant content, and technical difficulties.

Regardless of the setting, patient engagement and adherence to their recommended care plan is an important issue [39]. Our review confirms previous observations [22,25] that usage of web and computer-based interventions decreases over time, with the highest usage seen in the first month. This pattern is particularly concerning as the studies in this review had intervention periods of only 3-6 months, highlighting the challenge of engaging participants in a web-based environment even over the short term. The majority of studies in this review reported only login rates as a measure for usability [28,31,32]. Login rates capture only a broad measure of website usage, providing little information about engagement with an intervention [15]. Only one study [29] provided evidence of engagement, reporting both login rates and time spent on the site, with a consistent level of engagement observed across the 6-month intervention for all participants. This consistency may have been due to the provision of structured dietary education modules. In face-to-face settings, structured education has been demonstrated to be effective for assisting people with T2DM to improve glycemic control and overall health [2]. Various authors suggest social support may increase participation [10,21]; however, this was not demonstrated in our results, as even the more complex web-based interventions offering contact with peers [31,32], and/or health care professionals [29,31,32] contributed no difference in terms of achieving clinical outcomes.

myDIDeA [29] was the first study to focus solely on providing a web-based dietary behavior change intervention for people with T2DM in Malaysia, providing the longest intervention period of 6-months with a 12-month follow-up. The intervention group showed a 12.2-point greater DKAB score compared to the control group (Table 2). While no between-group changes were found for clinical outcomes (Table 3), within-group changes in fasting blood glucose and HbA_1c_ were only found in the intervention group at 12-months follow-up, which may indicate that web-based dietary interventions could continue to influence behavior change beyond the intervention period. Furthermore, myDIDeA had the lowest overall attrition rate, which may be explained by the provision of an intervention solely focused on diet. Since determining what to eat is a significant challenge for people with T2DM [2], this specific dietary focus and the structured nature of delivering the components may have encouraged clarity, fostering longer-term commitment. Additionally, myDIDeA integrated a user-centered approach during the development of the intervention. User-centered design is a human-factor engineering strategy for designing user-friendly platforms [40], a method that has been described in other web-based T2DM self-management interventions [41,42].

The Medical Research Council [39] suggests that best practice for complex interventions is using the best available evidence and appropriate theory. According to a systematic review and meta-analysis [43] of diet behavior change techniques in people with T2DM, the only intervention feature associated with significant reductions in HbA_1c_ was the application of a theoretical model or framework. However, the relationship between theoretical application and clinical outcomes has yet to be confirmed in web-based dietary behavior change interventions. For instance, the ANODE study [28] demonstrated statistically significant improvements in both dietary behavior and clinical outcomes, yet there was no theoretical basis for the development of the program. Rather it was a nutritional support tool developed by a private company.

Changes in glycemic control ranged from HbA_1c_ reductions of 0.3% [28] to 0.8% [30]. These results are clinically meaningful as previous research has demonstrated that for every 1% reduction in HbA_1c_, there is an associated risk reduction for heart attacks, microvascular complications, and deaths related to diabetes [3]. Achieving modest weight loss of ≥5% has been shown to improve glycemic control [2], a goal that was achieved by participants in the two studies that found statistically significant clinical outcomes between groups [28,30]. The VLCKD [30] produced the most significant results overall, with reductions in HbA_1c_, weight, and triglycerides. However, these results should be interpreted with caution due to the small study size. Furthermore, systematic reviews and meta-analysis of RCTs prescribing low-carbohydrate diets in people with T2DM [44], suggest that adherence to VLCKD interventions (<50 grams carbohydrates per day) are frequently poor and more difficult for people to maintain than a low-carbohydrate diet (50-130 grams carbohydrates per day), with no additional clinical benefits over a prescribed low-carbohydrate diet [45]. Overall, the results of this review demonstrated that, regardless of diet characteristics, participants who adhered to dietary recommendations showed improvements in their food choices and overall dietary quality, which improved clinical outcomes in most cases.

### Limitations and Future Research

Only five studies met the eligibility criteria, and these were heterogeneous in terms of dietary recommendations, focus on diet alone or with additional behavioral components, behavioral theories and models applied, and target population. The modes and methods of delivering the web-based interventions also differed, and the duration and follow up periods varied widely. While most studies report statistically significant improvements in dietary behavior change, the use of different dietary assessment measures made it difficult to compare study outcomes and generalize results. Web-based interventions could improve and expand reporting of website statistics to help inform patterns of participant behavior, and consideration of measuring adherence to diet as a means of determining if greater adherence leads to greater improvement in clinical outcomes is warranted. Web-based interventions could be a cost-effective way to provide education and support to individuals with T2DM, broadening access for a greater number of people, including those who have location or mobility constraints and cannot access face-to-face services. More research is needed to explore web-based dietary interventions for these diverse populations, including younger adults and the elderly. Web-based dietary interventions must be studied in larger cohorts, for longer durations, and with more clearly defined dietary recommendations. Studies must also explore intervention content, modes and methods of delivery, and whether these enhance participant engagement or contribute meaningfully to the expected outcomes.

### Conclusion

This review provided evidence that web-based interventions may be an effective way to improve dietary behavior in people with T2DM. The results also suggest improvements in glycemic control and clinical outcomes may be possible, although the studies in this review yielded inconsistent results. While this preliminary evidence showed promise of a positive effect, the small number of studies and the fact they are highly heterogeneous makes it difficult to draw any firm conclusions. The field requires more well-designed web-based dietary interventions that report dietary prescription and adherence in people with T2DM to confirm their effectiveness in optimizing dietary behavior and improving clinical outcomes.

## Appendix

Multimedia Appendix 1 Table 4. Intervention components, attrition, usability and modes and methods of delivery.

Multimedia Appendix 2 Table 5. Process evaluation measures.

## Abbreviations

ADA: American Diabetes Association
ANODE: Accompagnement Nutritionnel de l’Obésité et du Diabète par E­coaching
CASM/+: computer-assisted self-management/computer-assisted self-management plus social support
CINAHL: Cumulative Index of Nursing and Allied Health Literature
CVD: cardiovascular disease
DKAB: Dietary Knowledge, Attitude and Behavior
D-Net: Diabetes Network
HbA_1c_: hemoglobin A_1c_
mHealth: mobile health
myDIDeA: Malaysian Dietary Intervention for People with Type 2 Diabetes: An e-Approach
NCI: National Cancer Institute
PRISMA: Preferred Reporting Items for Systematic Reviews and Meta-Analyses
PROSPERO: International Prospective Register of Systematic Reviews
RCT: randomized controlled trial
RoB 2: Cochrane risk-of-bias tool for randomized trials
T2DM: type 2 diabetes mellitus
VLCKD: very low carbohydrate ketogenic diet

## References

[1] Kaiser AB, Zhang N, Der Pluijm WV (2018). Global Prevalence of Type 2 Diabetes over the Next Ten Years (2018-2028). Diabetes.

[2] American Diabetes Association (2019). Standards of Medical Care in Diabetes 2019. Diabetes Care (Supplement 1).

[3] Stratton IM, Adler AI, Neil HA, Matthews DR, Manley SE, Cull CA, Hadden D, Turner RC, Holman RR (2000). Association of glycaemia with macrovascular and microvascular complications of type 2 diabetes (UKPDS 35): prospective observational study. BMJ.

[4] Evert AB, Dennison M, Gardner CD, Garvey WT, Lau KHK, MacLeod J, Mitri J, Pereira RF, Rawlings K, Robinson S, Saslow L, Uelmen S, Urbanski PB, Yancy WS (2019). Nutrition Therapy for Adults With Diabetes or Prediabetes: A Consensus Report. Diabetes Care.

[5] Lopez JMS, Katic BJ, Fitz-Randolph M, Jackson RA, Chow W, Mullins CD (2016). Understanding preferences for type 2 diabetes mellitus self-management support through a patient-centered approach: a 2-phase mixed-methods study. BMC Endocr Disord.

[6] Pal K, Dack C, Ross J, Michie S, May C, Stevenson F, Farmer A, Yardley L, Barnard M, Murray E (2018). Digital Health Interventions for Adults With Type 2 Diabetes: Qualitative Study of Patient Perspectives on Diabetes Self-Management Education and Support. J Med Internet Res.

[7] Cassimatis M, Kavanagh DJ, Smith AC (2014). Perceived Needs for Supported Self-management of Type 2 Diabetes: A Qualitative Investigation of the Potential for a Web-based Intervention. Australian Psychologist.

[8] Booth AO, Lowis C, Dean M, Hunter SJ, McKinley MC (2013). Diet and physical activity in the self-management of type 2 diabetes: barriers and facilitators identified by patients and health professionals. Prim Health Care Res Dev.

[9] Kennedy M, Dunning T (2017). Diabetes education: essential but underfunded in Australia. Diabetes & Primary Care Australia.

[10] Pereira K, Phillips B, Johnson C, Vorderstrasse A (2015). Internet delivered diabetes self-management education: a review. Diabetes Technol Ther.

[11] Managing diabetes: improving services for people with diabetes. Commission for Healthcare Audit Inspection, London, UK.

[12] Australian Diabetes Educators Association. Workforce in Diabetes Education (Online).

[13] Chatterjee S, Davies MJ, Heller S, Speight J, Snoek FJ, Khunti K (2018). Diabetes structured self-management education programmes: a narrative review and current innovations. Lancet Diabetes Endocrinol.

[14] Odgers-Jewell K, Isenring EA, Thomas R, Reidlinger DP (2017). Group-based education for patients with type 2 diabetes: a survey of Australian dietitians. Aust J Prim Health.

[15] Karekla M, Kasinopoulos O, Neto DD, Ebert DD, Van Daele T, Nordgreen T, Höfer S, Oeverland S, Jensen KL (2019). Best Practices and Recommendations for Digital Interventions to Improve Engagement and Adherence in Chronic Illness Sufferers. European Psychologist.

[16] Li J, Parrott S, Sweeting M, Farmer A, Ross J, Dack C, Pal K, Yardley L, Barnard M, Hudda M, Alkhaldi G, Murray E (2018). Cost-Effectiveness of Facilitated Access to a Self-Management Website, Compared to Usual Care, for Patients With Type 2 Diabetes (HeLP-Diabetes): Randomized Controlled Trial. J Med Internet Res.

[17] Ramadas A, Quek KF, Chan CKY, Oldenburg B (2011). Web-based interventions for the management of type 2 diabetes mellitus: a systematic review of recent evidence. Int J Med Inform.

[18] Kitsiou S, Paré G, Jaana M, Gerber B (2017). Effectiveness of mHealth interventions for patients with diabetes: An overview of systematic reviews. PLoS One.

[19] Fu H, McMahon SK, Gross CR, Adam TJ, Wyman JF (2017). Usability and clinical efficacy of diabetes mobile applications for adults with type 2 diabetes: A systematic review. Diabetes Res Clin Pract.

[20] Nangrani N, Malabu U, Vangaveti V (2018). Outcomes of Telehealth in the Management of Type 2 Diabetes—A Systematic Review and Meta-analysis of Randomised Controlled Trials. Diabetes.

[21] Kebede MM, Zeeb H, Peters M, Heise TL, Pischke CR (2018). Effectiveness of Digital Interventions for Improving Glycemic Control in Persons with Poorly Controlled Type 2 Diabetes: A Systematic Review, Meta-analysis, and Meta-regression Analysis. Diabetes Technol Ther.

[22] Cotterez AP, Durant N, Agne AA, Cherrington AL (2014). Internet interventions to support lifestyle modification for diabetes management: a systematic review of the evidence. J Diabetes Complications.

[23] Moher D, Liberati A, Tetzlaff J, Altman DG (2009). Preferred reporting items for systematic reviews and meta-analyses: the PRISMA statement. PLoS Med.

[24] Neve M, Morgan PJ, Jones PR, Collins CE (2010). Effectiveness of web-based interventions in achieving weight loss and weight loss maintenance in overweight and obese adults: a systematic review with meta-analysis. Obes Rev.

[25] Pal K, Eastwood SV, Michie S, Farmer A, Barnard ML, Peacock R, Wood B, Edwards P, Murray E (2014). Computer-based interventions to improve self-management in adults with type 2 diabetes: a systematic review and meta-analysis. Diabetes Care.

[26] Pal K, Eastwood SV, Michie S, Farmer AJ, Barnard ML, Peacock R, Wood B, Inniss JD, Murray E (2013). Computer-based diabetes self-management interventions for adults with type 2 diabetes mellitus. Cochrane Database Syst Rev.

[27] Sterne JAC, Savović J, Page MJ, Elbers RG, Blencowe NS, Boutron I, Cates CJ, Cheng H, Corbett MS, Eldridge SM, Emberson JR, Hernán MA, Hopewell S, Hróbjartsson A, Junqueira DR, Jüni P, Kirkham JJ, Lasserson T, Li T, McAleenan A, Reeves BC, Shepperd S, Shrier I, Stewart LA, Tilling K, White IR, Whiting PF, Higgins JPT (2019). RoB 2: a revised tool for assessing risk of bias in randomised trials. BMJ.

[28] Hansel B, Giral P, Gambotti L, Lafourcade A, Peres G, Filipecki C, Kadouch D, Hartemann A, Oppert J, Bruckert E, Marre M, Bruneel A, Duchene E, Roussel R (2017). A Fully Automated Web-Based Program Improves Lifestyle Habits and HbA_1c_ in Patients With Type 2 Diabetes and Abdominal Obesity: Randomized Trial of Patient E-Coaching Nutritional Support (The ANODE Study). J Med Internet Res.

[29] Ramadas A, Chan CKY, Oldenburg B, Hussein Z, Quek KF (2018). Randomised-controlled trial of a web-based dietary intervention for patients with type 2 diabetes: changes in health cognitions and glycemic control. BMC Public Health.

[30] Saslow LR, Mason AE, Kim S, Goldman V, Ploutz-Snyder R, Bayandorian H, Daubenmier J, Hecht FM, Moskowitz JT (2017). An Online Intervention Comparing a Very Low-Carbohydrate Ketogenic Diet and Lifestyle Recommendations Versus a Plate Method Diet in Overweight Individuals With Type 2 Diabetes: A Randomized Controlled Trial. J Med Internet Res.

[31] Glasgow RE, Boles SM, McKay HG, Feil EG, Barrera M (2003). The D-Net diabetes self-management program: long-term implementation, outcomes, and generalization results. Prev Med.

[32] Glasgow RE, Kurz D, King D, Dickman JM, Faber AJ, Halterman E, Woolley T, Toobert DJ, Strycker LA, Estabrooks PA, Osuna D, Ritzwoller D (2012). Twelve-month outcomes of an Internet-based diabetes self-management support program. Patient Educ Couns.

[33] Ramadas A, Chan CKY, Oldenburg B, Hussien Z, Quek KF (2015). A web-based dietary intervention for people with type 2 diabetes: development, implementation, and evaluation. Int J Behav Med.

[34] Glasgow RE, Strycker LA, Kurz D, Faber A, Bell H, Dickman JM, Halterman E, Estabrooks PA, Osuna D (2010). Recruitment for an internet-based diabetes self-management program: scientific and ethical implications. Ann Behav Med.

[35] Glasgow RE, Christiansen SM, Kurz D, King DK, Woolley T, Faber AJ, Estabrooks PA, Strycker L, Toobert D, Dickman J (2011). Engagement in a diabetes self-management website: usage patterns and generalizability of program use. J Med Internet Res.

[36] Ramadas A, Quek KF, Chan CKY, Oldenburg B, Hussein Z (2011). Randomised-controlled trial of a web-based dietary intervention for patients with type 2 diabetes mellitus: study protocol of myDIDeA. BMC Public Health.

[37] McKay HG, Glasgow RE, Feil EG, Boles SM, Barrera MJ (2002). Internet-based diabetes self-management and support: Initial outcomes from the Diabetes Network project. Rehabilitation Psychology.

[38] Lie SS, Karlsen B, Oord ER, Graue M, Oftedal B (2017). Dropout From an eHealth Intervention for Adults With Type 2 Diabetes: A Qualitative Study. J Med Internet Res.

[39] Medical Research Council. Developing and evaluating complex interventions: Following considerable development in the field since 2006, MRC and NIHR have jointly commissioned an update of this guidance to be published in 2019 (Online).

[40] LeRouge C, Wickramasinghe N (2013). A review of user-centered design for diabetes-related consumer health informatics technologies. J Diabetes Sci Technol.

[41] Murray E, Sweeting M, Dack C, Pal K, Modrow K, Hudda M, Li J, Ross J, Alkhaldi G, Barnard M, Farmer A, Michie S, Yardley L, May C, Parrott S, Stevenson F, Knox M, Patterson D (2017). Web-based self-management support for people with type 2 diabetes (HeLP-Diabetes): randomised controlled trial in English primary care. BMJ Open.

[42] Yu CH, Parsons JA, Hall S, Newton D, Jovicic A, Lottridge D, Shah BR, Straus SE (2014). User-centered design of a web-based self-management site for individuals with type 2 diabetes - providing a sense of control and community. BMC Med Inform Decis Mak.

[43] Cradock KA, ÓLaighin G, Finucane FM, McKay R, Quinlan LR, Martin GKA, Gainforth HL (2017). Diet Behavior Change Techniques in Type 2 Diabetes: A Systematic Review and Meta-analysis. Diabetes Care.

[44] Huntriss R, Campbell M, Bedwell C (2018). The interpretation and effect of a low-carbohydrate diet in the management of type 2 diabetes: a systematic review and meta-analysis of randomised controlled trials. Eur J Clin Nutr.

[45] McArdle PD, Greenfield SM, Rilstone SK, Narendran P, Haque MS, Gill PS (2019). Carbohydrate restriction for glycaemic control in Type 2 diabetes: a systematic review and meta-analysis. Diabet Med.

