# Supplementary material for: Web-Based Interventions for Dietary Behavior in Adults With Type 2 Diabetes: Systematic Review of Randomized Controlled Trials
Source: J Med Internet Res. 2020 Aug 28;22(8):e16437. doi: 10.2196/16437 (PMC7486668; doi:10.2196/16437)
Supplement: Multimedia Appendix 1 [file jmir_v22i8e16437_app1.pdf]

Table 4. Intervention components, attrition, usability and modes and methods of delivery

Author/Date	Intervention components	Attrition rate = N participants	Website usability rates	Modes/methods of delivery
Ramadas et al. 2018	• 12 dietary lesson plans delivered in stage-tailored recommendations based on automated SOC score quiz<br>• encouraged to send questions to nutritionist via website	IG = 3; CG = 7	Average login per lesson was once per participant with an average 12 minutes on site	User login required. Lesson plans provided one after another and released every 2 weeks over the 6 month intervention period. Login reminders sent every 2 weeks when updates released, followed by text message if failed to login within 3 days.
Hansel et al. 2017	4 modules:<br>1. diet and physical activity (PA) self-monitoring<br>2. nutritional assessment<br>3. balanced diet menu generator<br>4. PA education and prescription program	IG = 20; CG = 10	First month of intervention 93% of participants logged in, dropped to one-third of participants in the final month	User login required. Required to complete one week of dietary monitoring before accessing other modules, then simultaneous access to 4 modules allowed. Asked to connect at least once per week.
Saslow et al. 2017	• Program delivered via email included video lessons, printable handouts and links to online resources such as recipes<br>• Mailed out physical ketone test kits<br>• Mindfulness-based eating	IG = 1; CG = 6	Not reported	Emailed new lesson content weekly for 16 weeks, then every 2 weeks for the remaining 16 weeks. Asked to measure ketones at least once per week.

	behavior awareness training via handouts<br>• From week 6 included PA and sleep quality prompts			
Glasgow et al. 2003	• Computer-mediated access to professional coach to give tailored dietary advice and help set goals<br>• Online interactive dietary assessment<br>• Online dietitian Q&A<br>• Online graphic self-monitoring for blood glucose and food diary<br>• Newsletters posted to website<br>• Activities to exchange diabetes-related info via peer directed forum monitored by health professionals<br>• Online introduction to diabetes-specific topics<br>• Provision of local restaurant lists with menu strategies	Overall attrition rate 18%, group numbers not reported	Users in the two intervention arms logged in on average 16.7-18.7 in first 1-3 months, which decreased to 5.3-6.7 in the last 7-10 months	User login required. Online dietary assessment at baseline, 3 months and 6 months. Newsletters posted to website every 2 months. Interaction with peers and professionals ad libitum.

Glasgow et al. 2012	CASM<br>• Tracking section of website for diet and blood glucose with graphical outputs<br>• Website also provided graphical outputs for cholesterol, SBP, HbA1c<br>• Community resources such as recipes, printable handouts<br>• Moderated forum<br>• Rotating quiz questions<br>• Periodic motivational calls from computer-based phone system<br>CASM+ received all of the components above, plus:<br>• 2 follow-up calls from interventionist<br>• Invitation to attend 120 min group visits for diabetes education from nutritionists and physicians	CASM = 69, CASM+ = 58;<br>CG = 18	Users in the two intervention arms logged in on average 10.6 times in month one, reduced to 4.36 times by month 6, to 2.5 times at 12 months	User login required. Self-administered initial goal setting and/or with assistance from staff member and receive immediate feedback. After 6 weeks participants created action plans, which were self-administered and/or collaboratively with interventionists, which could be revised as needed. Additional calls provided 2-8 weeks after initial visit.

CASM = computer-assisted self-management, CASM+ = computer-assisted self-management plus social support, CG = control group, HbA1c = Hemoglobin A1c, IG = intervention group, PA = physical activity, SBP = systolic blood pressure, SOC = Stages of Change Theory.

Web-based interventions for dietary behavior in adults with type 2 diabetes: a systematic review of randomized controlled trials

Jedha Dening, Sheikh Mohammed Shariful Islam, Elena George, Ralph Maddison
