# Supplementary material for: Web-Based Interventions for Dietary Behavior in Adults With Type 2 Diabetes: Systematic Review of Randomized Controlled Trials
Source: J Med Internet Res. 2020 Aug 28;22(8):e16437. doi: 10.2196/16437 (PMC7486668; doi:10.2196/16437)
Supplement: Multimedia Appendix 2 [file jmir_v22i8e16437_app2.pdf]

Table 5. Process evaluation measures

Author/Date	Adherence	Usability	Acceptability	Content/program satisfaction
Ramadas et al. 2018	Number of logins and time spent on site	Facilitating conditions and user-friendliness (rating 72%)	Attitude towards technology, anxiety, self-efficacy, and behavioral intention (rating 62%)	Performance expectancy, effort expectancy, and patient-centered factors (rating 64%)
Hansel et al. 2017	Website login rates	Not reported	Assessed in terms of program satisfaction	Automated post-intervention satisfaction questionnaire (92% of intervention group returned, 70% would recommend program)
Saslow et al. 2017	No process evaluation reported	Not reported	Not reported	
Glasgow et al. 2003	Delivery of intervention components and usage of website (pages viewed, areas of website visited, average logins, and timeframe), though only frequency of login was reported	Not reported	Not reported	Reported 100% implementation rate of online interactive dietary assessments but no official satisfaction rates reported
Glasgow et al. 2012	Website login rates	Not reported	Not reported	Not reported

Web-based interventions for dietary behavior in adults with type 2 diabetes: a systematic review of randomized controlled trials

Jedha Dening, Sheikh Mohammed Shariful Islam, Elena George, Ralph Maddison
